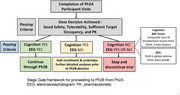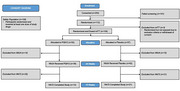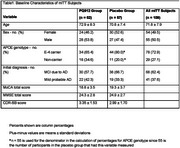# The VIVA‐MIND study: Topline Results from Phase 2 RCT of Varoglutamstat in Early AD

**DOI:** 10.1002/alz70859_105693

**Published:** 2025-12-26

**Authors:** Howard H. Feldman, Karen Messer, Jing Zhang, Natalie E Quach, Gabriel C Leger, Diane M. Jacobs, Steven D Edland, Jasmin Duehring, Andrew MacKelfresh, Alessandra Pol, Carolyn Revta, Jody‐Lynn Lupo, Archana Balasubramanian, Nicola Lama, Tanja Wassmann, Michael Schaeffer, Antje Meyer, Sylvia Schell‐Mader, Christine Wenzkowski, Frank Weber, ADCS VIVA‐MIND Study Group

**Affiliations:** ^1^ Alzheimer's Disease Cooperative Study, University of California San Diego, La Jolla, CA USA; ^2^ Department of Neurosciences, University of California San Diego, La Jolla, CA USA; ^3^ Shiley‐Marcos Alzheimer’s Disease Research Center, La Jolla, CA USA; ^4^ Alzheimer’s Disease Cooperative Study, University of California San Diego, La Jolla, CA USA; ^5^ UCSD Shiley‐Marcos Alzheimer's Disease Research Center, La Jolla, CA USA; ^6^ Department of Neurosciences, University of California San Diego, San Diego, CA USA; ^7^ Certara, Basel Switzerland; ^8^ Vivoryon Therapeutics NV, Halle Germany

## Abstract

**Background:**

Varoglutamstat (PQ912) is an oral small molecule inhibitor of glutaminyl cyclases which reduces the pyroglutamate formation of Aβ and CCL2. Preclinical and Ph1 trials support this Ph2 evaluation in early AD. Our objectives included selecting the highest safe and well‐tolerated dose of varoglutamstat with futility analysis at 24 weeks (Ph2A) followed by seamless evaluation of longer‐term safety and efficacy at 72 weeks at this dose (Ph2B).

**Method:**

Participants with biomarker confirmed early AD were randomized to varoglutamstat or placebo. A sequential design tested 3 descending doses (600mg, 300mg, 150mg BID) using a continuous Pocock safety boundary. A Stage Gate futility analysis of the first 180 subjects at 24 weeks was designed to guide the decision from Ph2A‐B (Figure 1). The Ph2B primary endpoint was CDR‐sum‐of‐boxes (CDR‐SB), with secondary endpoints CFC2, ABC score, spectral EEG, FAQ, ADAS‐Cog‐13, and NPI.

**Result:**

Table 1 presents baseline characteristics of the 109 randomized and dosed participants. The first dose cohort of varoglutamstat 600mg or placebo did not cross the safety boundary in Ph2A, supporting this dose selection for all participants thereafter. An abbreviated futility analysis was conducted after the trial was terminated prematurely for administrative reasons, with the provisional result of ‘halt enrollment’(yellow) (Figure 1). There were 74% of participants who completed week 24 and 31% week 72 (Figure 2). There were no significant differences between varoglutamstat and placebo in LS mean squares from baseline to week 72 in CDR‐SB (‐0.05 [95% CI ‐1.03, 0.92]), CFC2 (0.97 [95% CI ‐3.34, 5.28]), ABC score (0.10 [95% CI ‐0.10, 0.30]), FAQ (‐0.60 [95% CI ‐4.22, 3.01]), ADAS‐Cog‐13 (2.03 [95% CI ‐2.29, 6.36]); or NPI (‐3.09 [95% CI ‐7.81, 1.62]). At least one TEAE during treatment was reported in 84.9% varoglutamstat and 76.8% placebo with treatment discontinuations due to AE 11.3% varoglutamstat and 3.4% placebo. Severe TEAEs occurred with similar frequencies across arms. Frequency of SAEs/AESIs was 18.9/1.9% varoglutamstat versus 8.9%/5.4% placebo.

**Conclusion:**

Varoglutamstat was safe and well‐tolerated in this VIVA‐MIND early AD RCT. Efficacy assessment did not demonstrate any significant treatment benefits however, the available sample was limited. Biomarker results and PK/PD are pending.